# Syringomyelia-like syndrome in neuromyelitis optica spectrum disorder complicated with Sjogren’s syndrome: a case report

**DOI:** 10.1186/s12883-018-1170-9

**Published:** 2018-10-09

**Authors:** Xiangling Li, Zhengqi Lu, Yanqiang Wang

**Affiliations:** 1 Department of Nephrology, Department of Internal Medicine, Wei fang Medical University, Weifang, China; 2 Department of Neurology, The Third Affiliated Hospital of Sun Yat-sen University, Guangzhou, China; 3 Department of Neurology, The Affiliated Hospital of Wei fang Medical University, No 2428 Yuhe Road, Weifang, 261031 Shandong China

**Keywords:** Neuromyelitis optica spectrum disorder, Aquaporin-4 antibodies, Oligoclonal bands, Syringomyelia-like syndrome, Sjogren’s syndrome

## Abstract

**Background:**

Besides CSF-flow obstruction, syringomyelia is associated with inflammatory spinal cord lesions. However, syringomyelia-like syndrome concomitant with neuromyelitis optica spectrum disorder (NMOSD) and primary Sjogren’s syndrome (pSS) is extremely rare. Here, we would like to report a case of a patient with syringomyelia-like syndrome in NMOSD complicated with Sjogren’s Syndrome.

**Case presentation:**

A 64-old male Han Chinese, presented with three episodes of acute demyelinating processes in the central nervous system within 5 years. Firstly, he presented with ascending left lower extremity weakness and numbness, and initially progressive loss of vision in the right eye before 5 years, and subsequently in the right eye 2 months later. High dose corticosteroid therapy was prescribed for this attack. Second, he suffered from refractory gastrointestinal symptoms.

such as nausea, vomiting, abdominal pain and early satiety. After the second episode, he received long-term azathioprine and prednisone treatment in low dosages. Six months before admission, he developed the lower back pain and numbness in lower limbs, and urinary incontinence. This time, he complained of acute onset of right lower limb paralysis, paresthesia and urinary incontinence. MRI of the spine revealed a syringomyelia extending from the C7 to T4 levels with serum positive anti-aquaporin-4 antibodies (AQP4-Ab) (indirect immunofluorescence on AQP4 transfected cells). he was serologically positive for both anti-Sjögren’s syndrome-related antigen A and B antibodies and there was reduced salivary flow on scintigraphy. Lip salivary gland (LSG) biopsies were graded (grade four lymphocytic infiltration) according to the Chisholm and Mason classification system and by morphometric analysis. And finally, diagnosed as syringomyelia-like syndrome in NMOSD complicated with Sjogren’s syndrome.

**Conclusions:**

Although extremely rare, This index patient highlights that syringomyelia could be associated with underlying NMOSD and pSS, and autoimmune disorders should be considered in the initial differential diagnosis, This is very helpful for the therapeutic implications and evaluating curative effect.

## Background

Syringomyelia is a chronic, progressive and degenerative disorder, characterized by a cystic or cavitary formation within the spinal cord. The exact pathogenesis and development of syringomyelia is uncertain. Syringomyelia coexisting with multiple sclerosis (MS) has been described in various case reports/series. However, literatures describing syringomyelia coexisting with neuromyelitis optica spectrum disorder (NMOSD) and primary Sjogren’s syndrome (pSS) are extremely rare, and only a few syringomyelia in NMOSD have been reported [1–5]. We report a case of syringomyelia-like syndrome coexisting with NMOSD and pSS.

## Case presentation

**Fig. 1:**
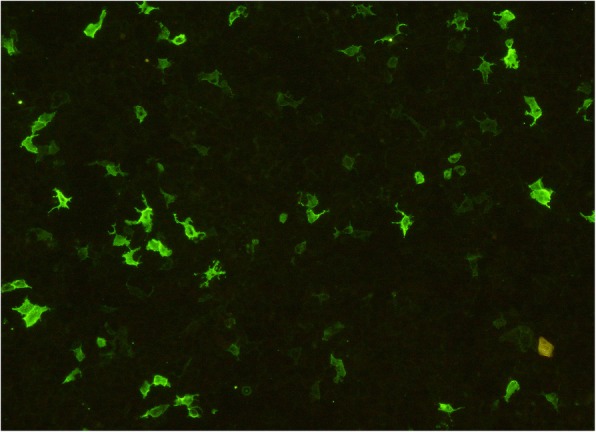
Serum anti-aquaporin-4 antibody CBA

**Fig. 2:**
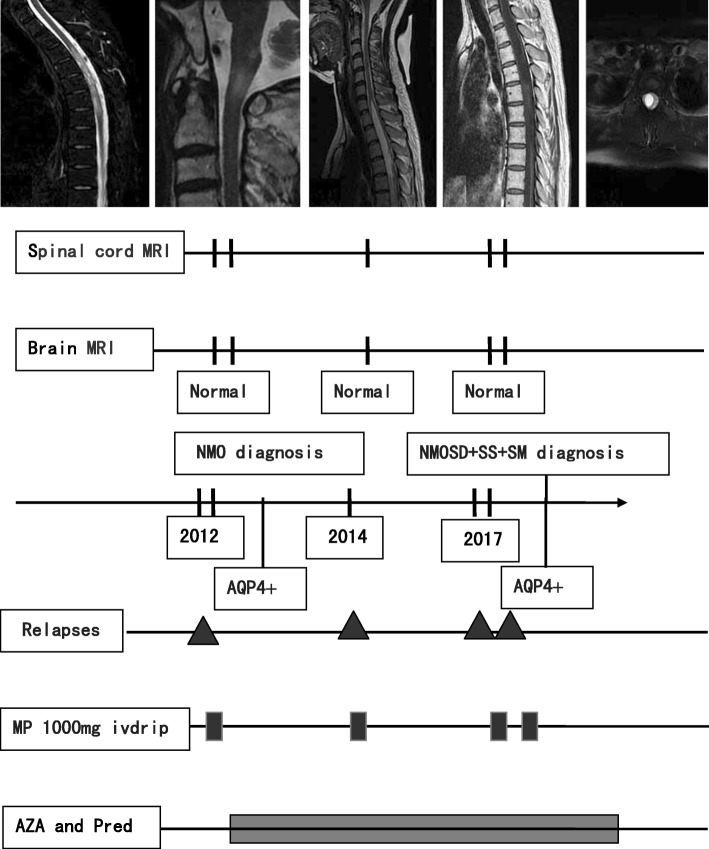
Disease course, MRI scans and therapy in the SM-like syndrome in NMOSD complicated with SS. The figure depicts clinical relapses (triangle), MRI findings (T2WI, T2 fs FSE, T2WI, T1WI, T2 fs FSE), and therapy data (methyl prednisolone (MP) 1000 mg ivdrip. 3–5 daily infusions; prednisone (pred) 60 mg orally daily with gradual taper; Azathioprine(AZA) was increased from 50 mg to 100 mg daily. AQP: aquaporin, AZA: azathioprine, FLAIR: fluid attenuated inversion recovery, MP: methyl prednisolone, pred: prednisone, MRI: magnetic resonance imaging, NMOSD: neuromyelitis optica spectrum disorder, SM: syringomyelia, SS: Sjogren syndrome

**Fig. 3:**
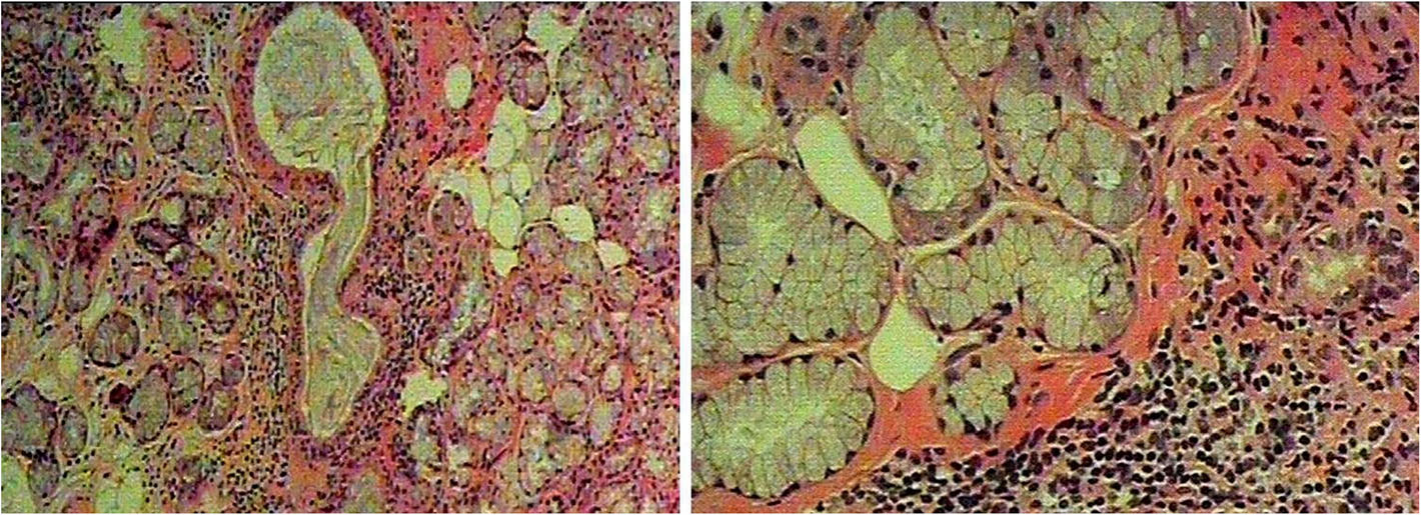
Histological analysis of salivary gland biopsies. Biopsies were stained using hematoxylin-eosin, Magnification: 400×, A and B are pointing to lymphocytic and plasma cell infiltration, loss of salivary gland parenchyma

A 64-old male Han Chinese was admitted due to acute onset of the right lower limb paralysis, paresthesia and urinary incontinence, preceded by six-month history of lower back pain and numbness in lower limbs, and urinary incontinence. Five years prior to the current presentation, he had the ascending left lower extremity weakness and numbness, but progressed extremely rapidly (in five days) resulting in gait instability, band-like sensory deficits, and accompanied by progressive loss of vision in the left eye initially, subsequently developed loss of vision in the right eye 2 months later. For 3 years, he had experienced refractory gastrointestinal symptoms, including nausea, vomiting, epigastric pain and early satiety. No history of skin rashes, photosensitivity, arthralgia, hair loss, oral ulcers, and recurrent fever, joint arthritis and erythema nodosum was identified. On specific questioning, he experienced a sensation in both eyes and a dry mouth since the past 2 months. This time, His vision was normal with no visual field defects or double images, and no optic atrophy or optic neuritis was detected on fundoscopy. Physical examination revealed normal vital signs, heart, lungs and abdominal examination. Cranial nerve examination was normal. Sensory examination revealed loss of pain sensation and tactile sensation (grade 1), displayed deficits in proprioception and touch sensation in the lower extremities (grade 1), Motor examination showed motor strength grade 1/5 and 4/5 in the right and left lower limb right respectively, Pain and temperature sensation was diminished to T4, light touch was mildly impaired to T4, vibration decreased to the knee on the right. Abdominal reflexes were absent, and tendon reflexes were equivocal. Babinski’s sign were present bilaterally, Kernig’s sign and Brudzinski’s sign were negative. Full blood counts, electrolytes, complement, kidney and liver functions, thyroid function, homocystein, Vitamin B12 Vitamin D, Vitamin B1, Complement C3, Complement C4, urinalysis results were all within normal limits. Other antibodies including rheumatoid factor (RF), anti-streptolysin “O”, thyroid-associated antibodies, ENA, anti-Sm, anti-Scl70, anti-Jo-1, anti-dsDNA, anti-centromere, anti-nucleosome, anti-histone, anti-ribosomal P protein antibodies, c-ANCA, p-ANCA, Anti-dsDNA were negative, Other laboratory datas including Lyme IgM, IgG, IgA, VDRL, HSV, VZV, CMV, EBV, HBV, HCV, HIV in serum and CSF were negative. Angiotensin-converting enzyme (ACE) were negative, Kveim test was negative. Abnormal laboratory findings included ESR 70 mm (< 20 mm), CRP 46 mg/l (< 20 mg/l), ANA + 1:640 [< 1:160; indirect immunofluorescence (IIF) on Hep2 cells], Anti-Sjögren’s syndrome A (SSA)/Ro, anti-Sjögren’s syndrome B (SSB)/La 1:4 [(0); double immunodiffusion assay]. Serum anti-aquaporin-4 antibody 1:320 [(0) (AQP4-Ab; AQP4-Ab was detected by indirect immunofluorescence staining using human AQP4 transfected HEK 293 cells, by a cellbased assay; CBA), This had been recommended in International consensus diagnostic criteria for neuromyelitis optica spectrum disorders)] (Fig. 1). Cerebrospinal fluid analysis revealed opening pressure 120 mmH2O (70–180 mmH2O), white cell count 13 cells/μl (0–5 cells/μl), total protein: 0.26 g/l (0.15–0.45 g/l), glucose: 3.76 mmol/L (2.5–4.4 mmol/L), chloride: 119.4 mmol/L (120–130 mmol/L). Cerebrospinal fluid analysis showed negative oligoclonal bands (OCB) by isoelectric focusing (IEF) on agarose gels followed by immunoblotting using an IgG-specific antibody staining. Schirmer’s I test showed normal lacrimal flow (> 15 mm at 5 min). CT chest/abdomen/pelvis was done which did not revealed bilateral hilar and mediastinal lymphadenopathy. Brain magnetic resonance imaging (MRI) did not show pathologic lesions. Spine MRI revealed syringomyelia from C7 to T4 (Fig. 2). Salivary scintigraphy showed bilateral submandibular and parotid gland excretion were impaired and bilateral submandibular gland uptake were slightly impaired. Lip biopsies were performed, the consisting of two lesions had exhibited greater than 50 lymphocytes in 4 cm3 along with focal lymphoctic infiltration, which was suggestive of pSS (Fig. 3).

## Discussion and conclusion

Neuromyelitis optica spectrum disorder (NMOSD) is an autoimmune inflammatory disease of the central nervous system (CNS) associated with antibodies against aquaporin 4 (AQP4), the target antigen of NMO-IgG was confirmed to be AQP4, the water channel protein which mainly expressed on astrocytic foot processes at the optic nerves, spinal cord, and certain areas of the brain. The cervical and thoracic cords are the most frequent sites involved in NMOSD, resulting in subsequent motor and sensory deficits presentations. Similarly, the common signs and symptoms of syringomyelia include muscle weaknesses, absence of pain, temperature sensations, and these symptoms often fluctuate in severity and duration, vary widely depending on the extent and the location of the syrinx inside the spinal to develop slowly but also occur abruptly [6, 7]. Many cases of syringomyelia have been reported as an incidental finding in multiple sclerosis (MS) or NMO [2, 8–10]. Syringomyelia.

during the course of the disease in patients presenting with clinically definite MS has been described in 4.5% of MS patients [8, 10]. However, there is only limited information regarding syringomyelia and NMOSD.

We evaluated many possible causes of syringomyelia in our patient. Finally diagnosis of the syringomyelia coexisting with NMOSD and pSS was confirmed. Other causes of myelopathy was excluded neurosarcoidosis sarcoidosis [11, 12], Behcet’s disease, vitamin B12 hypovitaminosis, and so on. The evaluation revealed a syringomyelia throughout the thoracic cord, from the C7 to T4 levels with anti-aquaporin-4 antibodies(NMO-IgG is a disease-specific autoantibody against AQP4 for NMOSD)positive and oligoclonal bands negative [13, 14]. Anti-Sjogren’s syndrome A and B antigen antibodies were positive, and there was reduced salivary flow on scintigraphy. Salivary gland biopsy showed lymphocytic infiltration. Sjogren’s syndrome met the diagnostic criteria of the 1968 Chisholm & Mason [15]. the clinical, serological, pathological biopsy and image features were fully consistent with the diagnosis of syringomyelia coexisting with NMOSD and pSS.

Growing evidence suggests autoimmune diseases more likely represent coexistent NMOSD rather than neurological complications of them. The wide spectrum of neurological manifestations in pSS have been reported, the prevalence of neurological symptoms of pSS range from 8.5 to 70%. However, Central nervous system (CNS) involvement is not common in PSS, approximately 2 to 25%, vary in presentation from focal, multifocal to additive or progressive, accompanying with a clinical course of fixed or cumulative deficits [16, 17]. The occurrence of myelopathy in pSS appears to be far from exceptional. Spinal cord involvement counld develop severe acute syndrome (acute transverse myelitis) or chronic progressive myelopathy [19–22]. Acute transverse myelitis is the most common form of spinal cord involvement in the course of pSS. The spectrum of clinical symptoms are diverse, relating to the region of the lesion. Our patient showed both prior acute attack and chronic progressive condition. pSS presenting as chronic progressive myelopathy, although rare, has previously been reported [19–22].

The exact mechanism of syringomyelia formation in the setting of NMOSD and Sjögren syndrome remained unclear [18, 23]. Increasing evidence about AQP4-IgG antibody binding to astrocytic AQP4 is involved in the pathogenesis of NMOSD, which causes cascade activation through complement dependent cytotoxicity (CDC), the infiltration of inflammatory cells, blood brain barrier disruption and oligodendrocyte injury and demyelination. Therefore, inflammatory lesions in NMOSD tend to be localized in site of high AQP4 expression, and in central gray matter. The pathogenesis of the gray matter damage is associated with primary selective area of high AQP4 expression, and speculate that anti-aquaporin 4 antibody play a crucial role in development of ‘syringomyelia-like’ syndrome, which causes blood brain barrier disruption, inflammation, necrosis of astrocytes and oligodendroglias, demyelination, and interferes with the circulation of CSF [2, 8, 10]. Other possible reasons include: interaction between the autoantibodies and the multiple targets tissues, shared common genetic, antigens and/or environmental factors which trigger an immune response cross-reacting, immunologic regulation disorder between NMOSD and pSS.

Therefore, Occasional previous reports have indicated that a fluid-filled syrinx-like cavity in the cord may be seen during the acute phase of myelitis in NMOSD. Futhermore, the fluid disappears on follow-up MRI and may not reappear during a subsequent attack of myelitis. This type of fluid accumulation is quite different from the usual type of chronic syringomyelia which is probably related in most cases to obstruction or impairment of CSF flow and not associated with myelitis. Syringomyelia is a consequence of the inflammatory demyelinating pathology or a part of clinical features of the NMOSD with pSS or a coincidental finding in a patient presenting with acute myelitis. Given the limited literature available, further research is necessary to elucidate the relationship between syringomyelia and NMOSD. Especially, long-term follow-up is crucial to clarify the spinal cord fluid in this patient which is transient or chronic. Since NMOSD and pSS coexist together, the diagnosis either condition should warrant further evaluation for the other condition due to potential treatment consideration [9].

This case report illustrates that ‘syringomyelia-like’ syndrome could be associated with underlying NMOSD and pSS. This finding may further emphasize the necessity and the importance of investigating for autoimmune disorders in ‘syringomyelia-like’ syndrome and subsequent therapeutic implications.

## Abbreviations

AQP4-Ab: Aquaporin-4 antibodies
MS: Multiple sclerosis
NMOSD: Neuromyelitis optica spectrum disorder
pSS: Sjogren’s syndrome
